# Soluble lectin‐like oxidized low‐density Lipoproteinreceptor‐1 and recurrent stroke: A nested case–control study

**DOI:** 10.1111/cns.13932

**Published:** 2022-07-31

**Authors:** Anxin Wang, Xue Tian, Jie Xu, Hao Li, Qin Xu, Pan Chen, Xia Meng, Yongjun Wang

**Affiliations:** ^1^ Department of Neurology Beijing Tiantan Hospital, Capital Medical University Beijing China; ^2^ China National Clinical Research Center for Neurological Diseases Beijing Tiantan Hospital, Capital Medical University Beijing China; ^3^ Department of Epidemiology and Health Statistics School of Public Health, Capital Medical University Beijing China

**Keywords:** nested case–control study, prognosis, recurrent stroke, soluble lectin‐like oxidized low‐density lipoproteinreceptor‐1

## Abstract

**Main problem:**

The prognostic value of soluble lectin‐like oxidized low‐density lipoproteinreceptor‐1 (sLOX‐1) for stroke was unclearly. This study aimed to investigate the association between sLOX‐1 and recurrent stroke in patients with acute ischemic stroke (AIS) or transient ischemic attack (TIA).

**Methods:**

Data were obtained from the Third China National Stroke Registry. Eligible cases consisted of 400 patients who developed recurrent stroke within 1‐year follow‐up, 800 controls were selected using age‐ and sex‐matched with a 1:2 case–control ratio. Conditional logistic regressions were used to evaluate the association between sLOX‐1 and recurrent stroke.

**Results:**

Among 1200 patients included in this study, the median (interquartile range) of sLOX‐1 was 247.12 (132.81–413.58) ng/L. After adjustment for conventional confounding factors, the odds ratio with 95% confidence interval in the highest tertile versus the lowest tertile was 2.23 (1.61–3.08) for recurrent stroke, 2.31 (1.64–3.24) for ischemic stroke, 2.30 (1.66–3.19) for combined vascular events within 1‐year follow‐up. Furthermore, the addition of sLOX‐1 to a conventional risk model had an incremental effect on predictive value for recurrent stroke (C‐statistics 0.76, *p* < 0.0001; integrated discrimination improvement 13.38%, *p* < 0.0001; net reclassification improvement 55.39%, *p* < 0.0001). Similar results were observed when the timepoint was set up as 3 months. Subgroup analysis showed the association between higher sLOX‐1 and recurrent stroke was more pronounced in patients with a history of stroke (*p* for interaction = 0.0062).

**Conclusions:**

sLOX‐1 was positively associated with the risk of recurrent stroke, which may be a candidate biomarker to improve risk stratification of recurrent stroke.

## Graphical abstract

The prognostic value of soluble lectin‐like oxidized low‐density lipoproteinreceptor‐1 (sLOX‐1) for stroke was unclearly. Based on the Third China National Stroke Registry, we conducted the nested case–control study with 1200 patients with acute ischemic stroke or TIA (400 cases and 800 controls) to investigate the association between sLOX‐1 and recurrent. The results showed that sLOX‐1 was positively associated with the risk of recurrent stroke, which may be a candidate biomarker to improve risk stratification of recurrent stroke.

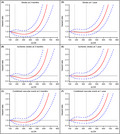

## 1 INTRODUCTION

Lectin‐like oxidized low‐density lipoprotein receptor‐1 (LOX‐1) is a scavenger receptor for oxidized low‐density lipoprotein (oxLDL) identified in endothelial cells.^1^, ^2^ Activation of LOX‐1 in endothelial cells induces various changes relevant to endothelia dysfunction, reduction in the release of nitric oxide, and induction of the expression of monocyte chemoattractant protein 1 and adhesion molecules, which lead to the development of atherosclerosis.^3^, ^4^, ^5^ Animal studies showed that deletion of LOX‐1 in mice preserves endothelial function and leads to reduction in atherogenesis.^6^ Basal expression of LOX‐1 is very low and upregulation of endothelial LOX‐1 is induced via oxLDL in proatherogenic conditions.^7^ A soluble form of LOX‐1 (sLOX‐1) is present in the circulation.^8^ sLOX‐1 is released from cells after proteolytic cleavage of membrane‐bound LOX‐1, and it has been assumed that sLOX‐1 reflects the cellular expression of LOX‐1.^9^, ^10^ Several reports showed the levels of sLOX‐1 were higher in those with acute stroke than in controls, suggesting that high levels of sLOX‐1 can be useful as diagnostic biomarker for acute stroke.^7^, ^11^ Additionally, high sLOX‐1 levels were also reported to be associated with an increased risk of adverse outcomes for patients with coronary heart disease.^12^ However, the clinical implications of serum sLOX‐1 levels in the prognosis of acute ischemic stroke (AIS) or transient ischemic attack (TIA) have not been clarified.

To address this knowledge gap, we conducted a nested case–control study using data from the China National Stroke Registry (CNSR‐III) to investigate the association between sLOX‐1 and recurrent stroke in patients with AIS or TIA.

## 2 METHODS AND MATERIALS

### 2.1 Study Population

**FIGURE 1:**
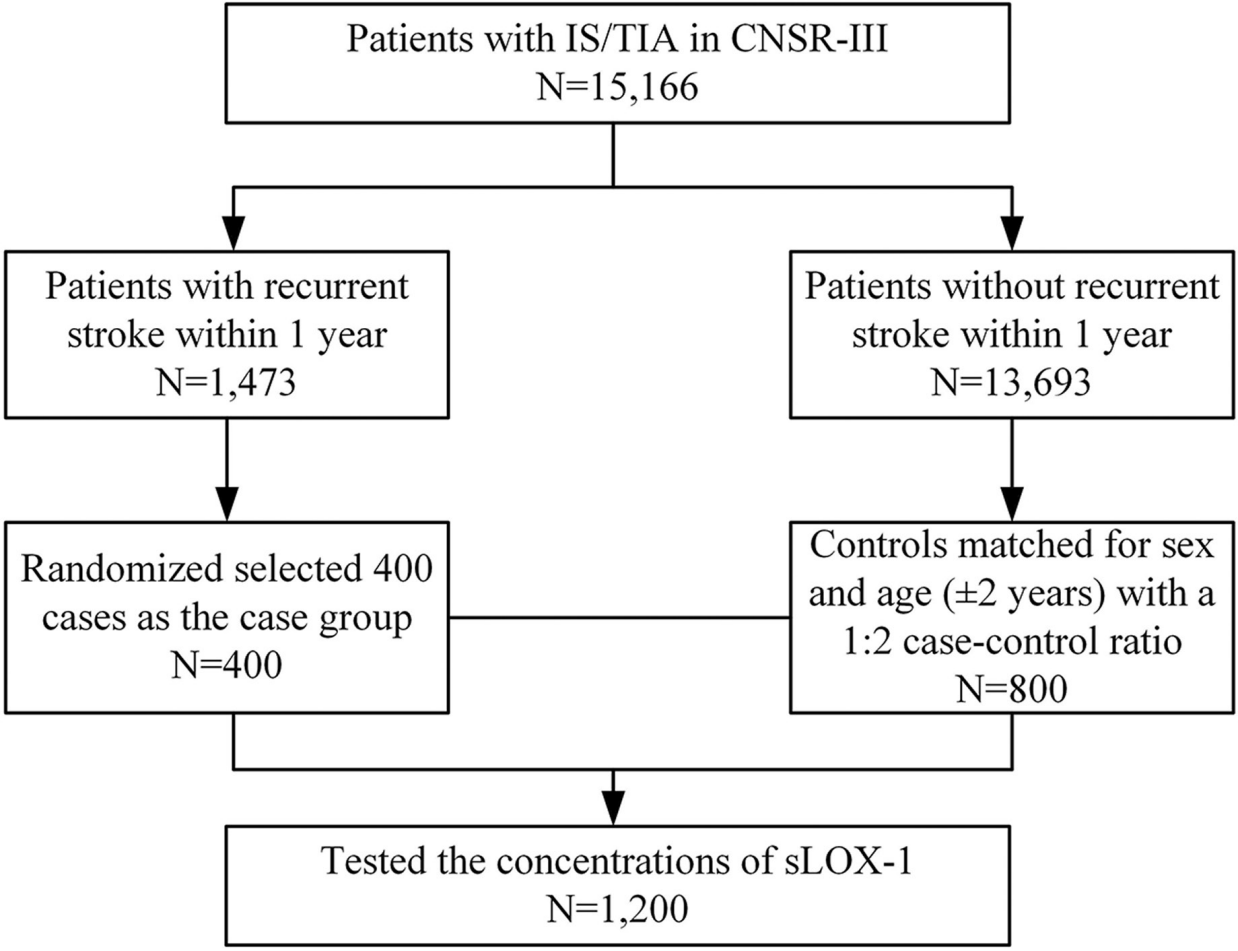
The flowchart of the study. Abbreviations: CNSR‐III = the Third China National Stroke Registry; IS = ischemic stroke; sLOX‐1 = Soluble lectin‐like oxidized low‐density lipoprotein receptor‐1; TIA = transient ischemic attack

Data were obtained from the CNSR‐III, which is a nationwide prospective registry that included patients with AIS or TIA presented to hospitals between August 2015 and March 2018 in China. The detailed design of the CNSR‐III has been previously described elsewhere.^13^ Participants were consecutively enrolled if meeting the following criteria: (1) age > 18 years old, (2) diagnosis of IS or TIA within 7 days, and (3) informed consent from participant or legally authorized representative. A total of 15,166 participants were enrolled from 201 study sites. After 1 year follow‐up, we identified 1473 cases of recurrent stroke. Among which, 400 cases were randomly selected as the case group. Controls were randomized selected and matched by age (±2 years) and sex at a 1:2 case–control ratio. Finally, a total of 1200 patients (400 cases and 800 controls) tested the concentrations of sLOX‐1 and were enrolled in the current study (Figure 1). The protocol of the CNSR‐III was approved by the Ethics Committee of Beijing Tiantan Hospital and all participating centers. All participants or their representatives provided written informed consents before being enrolled in the trial.

### 2.2 Measurement of sLOX‐1

Fasting blood samples were collected within 24 h of admission. Serum and plasma specimens were extracted and transported through cold chain to the core laboratory in Beijing Tiantan Hospital. All specimens were stored at −80°C until sLOX‐1 was detected centrally and blindly. The concentrations of sLOX‐1 were measured on anti‐analyzers by using 2 monoclonal antihuman LOX‐1 antibody (TS 92) as described previously.^12^

### 2.3 Assessment of Outcomes

The follow‐up procedure of CNSR‐III has been described previously. Patients were followed up by face‐to‐face or telephone interview at 3, 6 months, and 1 year by trained research personnel. Confirmation of stroke was sought from the treating hospital. Suspected recurrent stroke without hospitalization was judged by an independent end point judgment committee. The outcome was recurrent stroke (ischemic or hemorrhagic), ischemic stroke, and combined vascular events (including ischemic stroke, hemorrhagic stroke, myocardial infraction, or vascular death) within 3 months and 1 year.

### 2.4 Baseline Data Collection

The baseline data were collected by trained research coordinators though a direct interview or medical records, including age, sex, body mass index (calculated as the weight in kilograms divided by the square of the height in meters), time from onset of symptoms to admission, medical history of hypertension, diabetes, dyslipidemia, stroke or TIA, atrial fibrillation/flutter, peripheral vascular disease, heart failure, stroke type (IS or TIA), TOAST (Trial of Org 10,172 in Acute Stroke Treatment), medication in hospital, National Institutes of Health Stroke Scale score, and laboratory test at admission.

### 2.5 Statistical analysis

Normality of continuous variables was checked by Kolmogorov–Smirnov normality test. Continuous variables were expressed as median with interquartile range because of skewed distribution, and categorical variables were expressed as frequency with percentages. Participants were divided into three categories by tertiles of sLOX‐1. The nonparametric Wilcoxon or Kruskal–Wallis test was used to compared group differences for continuous variables, and Chi‐square test was used for categorical variables.

Multivariable conditional logistic regression analyses were performed to examine the association of sLOX‐1 with recurrent stroke. Variables were adjusted in the multivariable analyses if established as a traditional predictors for recurrent stroke or associated with sLOX‐1 in univariate analysis with a value of *p* < 0.10, including BMI, history of stroke, heart failure, stroke subtype, TOAST, antiplatelet agents, anticoagulant agents, high‐density lipoprotein cholesterol (HDL‐C), and fasting blood glucose (FBG). Unadjusted and adjusted odds ratios (ORs) and their 95% confidence intervals (CIs) were calculated. Trend tests were performed in the regression models after the median of sLOX‐1 values of each tertiles were entered into the model and treated as a continuous variable. In addition, we used restricted cubic splines to examine the shape of the association between sLOX‐1 and outcomes with four knots (at the 5th, 35th, 65th, and 95th percentiles). The reference point for sLOX‐1 was the median of the reference (the lowest tertile), and the OR was adjusted for all confounding variables mentioned above. Furthermore, we used C statistics, integrated discrimination improvement (IDI), and net reclassification index to evaluate the incremental predictive value of sLOX‐1 beyond conventional risk factors. To test the robustness of our findings, several additional analyses were performed. First, to minimize potential reverse causation, we excluded recurrent stroke, which occurred within the first month in the sensitivity analysis. Second, to examine whether the association between sLOX‐1 and recurrent stroke differs in different population, subgroup analyses were performed by stratifying variables, which were adjusted in the multivariable analysis with recurrent stroke at 1 year as the outcome of interest. The significance of the interaction between stratified variables and sLOX‐1 was tested using likelihood ratio test.

Overall, a two‐sided value of *p* < 0.05 was considered statistically significant. All analyses were performed with sas software version 9.4 (SAS Institute Inc).

## 3 RESULTS

### 3.1 Baseline characteristics

Among 1473 patients with recurrent stroke within 1 year, we randomly selected 400 cases into the current study. The baseline characteristics between excluded and included patients were well‐balanced (Table S1). A total of 400 cases and 800 controls were enrolled on the study, the median age was 64.00 (interquartile range: 55.00–72.50) years, and 798 (66.5%) were men. Baseline characteristics between cases and controls are presented in Table 1. Compared with controls, cases had higher a higher BMI, a higher proportion of ischemic stroke, large‐artery atherosclerosis, were more likely to take antiplatelet and anticoagulant agents, and had a high FBG level. Individuals with a higher sLOX‐1 level had a higher prevalence of heart failure, a higher proportion of large‐artery atherosclerosis stroke, were more likely to take hypoglycemic agents, and had a higher HDL‐C levels, compared with those with a lower sLOX‐1 level (Table S2).

**TABLE 1:** Baseline characteristics between cases and controls

Characteristics	Overall	Cases (*n* = 800)	Controls (*n* = 400)	*p* value
Age, year	64.00 (55.00–72.50)	64.00 (55.00–72.00)	64.00 (55.00–73.00)	0.9910
Men, *n* (%)	798 (66.5)	532 (66.50)	266 (66.50)	1.0000
Body mass index, kg/m^2^	24.49 (22.59–26.71)	24.29 (22.53–26.57)	24.92 (22.81–27.25)	0.0462
Time from onset of symptoms to admission ≥ 24 h, *n* (%)	482 (40.17)	332 (41.50)	150 (37.50)	0.1827
Medical History, *n* (%)				
Hypertension	774 (64.50)	509 (63.63)	265 (66.25)	0.3704
Diabetes mellitus	344 (28.67)	225 (28.13)	119 (29.75)	0.5573
Dyslipidemia	104 (8.67)	68 (8.50)	36 (9.00)	0.7717
Stroke or TIA	304 (25.33)	189 (23.63)	115 (28.75)	0.0543
Atrial fibrillation/flutter	100 (8.33)	65 (8.13)	35 (8.75)	0.7119
Peripheral vascular disease	13 (1.08)	11 (1.38)	2 (0.50)	0.1675
Heart failure	10 (5.41)	7 (5.74)	3 (4.76)	0.7809
Stroke type/Subtype, *n* (%)				
Ischemic stroke	1129 (94.08)	739 (92.38)	390 (97.50)	0.0004
TIA	71 (5.92)	61 (7.63)	10 (2.50)	
TOAST, *n* (%)				
Large‐artery atherosclerosis	344 (28.67)	204 (25.50)	140 (35.00)	0.0006
Cardioembolism	94 (7.83)	60 (7.50)	34 (8.50)	
Small‐vessel occlusion	215 (17.92)	156 (19.50)	59 (14.75)	
Other determined etiology	11 (0.92)	4 (0.50)	7 (1.75)	
Undetermined etiology	536 (44.67)	376 (47.00)	160 (40.00)	
Medication in hospital, *n* (%)				
Cholesterol‐lowering agents	1152 (96.08)	769 (96.13)	383 (95.99)	0.9096
Antihypertensive agents	575 (47.96)	380 (47.50)	195 (48.87)	0.6540
Hypoglycemic agents	362 (30.19)	231 (28.88)	131 (32.83)	0.1596
Antiplatelet agents	1156 (96.41)	779 (97.38)	377 (94.49)	0.0113
Anticoagulant agents	150 (12.51)	81 (10.13)	69 (17.29)	0.0004
NIHSS score on admission	4 (2–6)	3 (1–6)	4 (2–7)	0.0007
Laboratory tests				
Total cholesterol, mmol/L	3.88 (3.32–4.61)	3.85 (3.36–4.58)	3.95 (3.23–4.65)	0.4710
LDL‐C, mmol/L	2.22 (1.76–2.88)	2.19 (1.78–2.83)	2.29 (1.74–2.95)	0.2676
HDL‐C, mmol/L	0.95 (0.78–1.12)	0.96 (0.79–1.14)	0.93 (0.78–1.09)	0.0682
Triglyceride, mmol/L	1.34 (1.02–1.8)	1.35 (1.04–1.8)	1.31 (1.00–1.82)	0.4274
Fasting blood glucose, mmol/L	5.56 (4.90–7.12)	5.44 (4.85–6.91)	5.81 (5.08–7.65)	0.0034
eGFR, ml/min/1.73 m^2^	91.59 (80.74–100.69)	91.97 (80.65–100.42)	90.89 (80.77–101.25)	0.7987
Hs‐CRP, mg/dl	3.31 (1.47–6.87)	3.30 (1.40–6.10)	3.41 (1.60–7.92)	0.3035
sLOX, ng/L	247.12 (132.81–413.58)	229.87 (125.09–358.59)	301.45 (160.09–573.21)	<0.0001

Abbreviations: eGFR = estimated glomerular filtration rate; hs‐CRP, high sensitivity C‐reactive protein; NIHSS = The National Institutes of Health Stroke Scale; sLOX‐1 = Soluble lectin‐like oxidized low‐density lipoprotein receptor‐1; TIA = transient Ischemic Attack; TOAST = Trial of Org 10,172 in Acute Stroke Treatment.

### 3.2 sLOX‐1 and recurrent stroke

**FIGURE 2:**
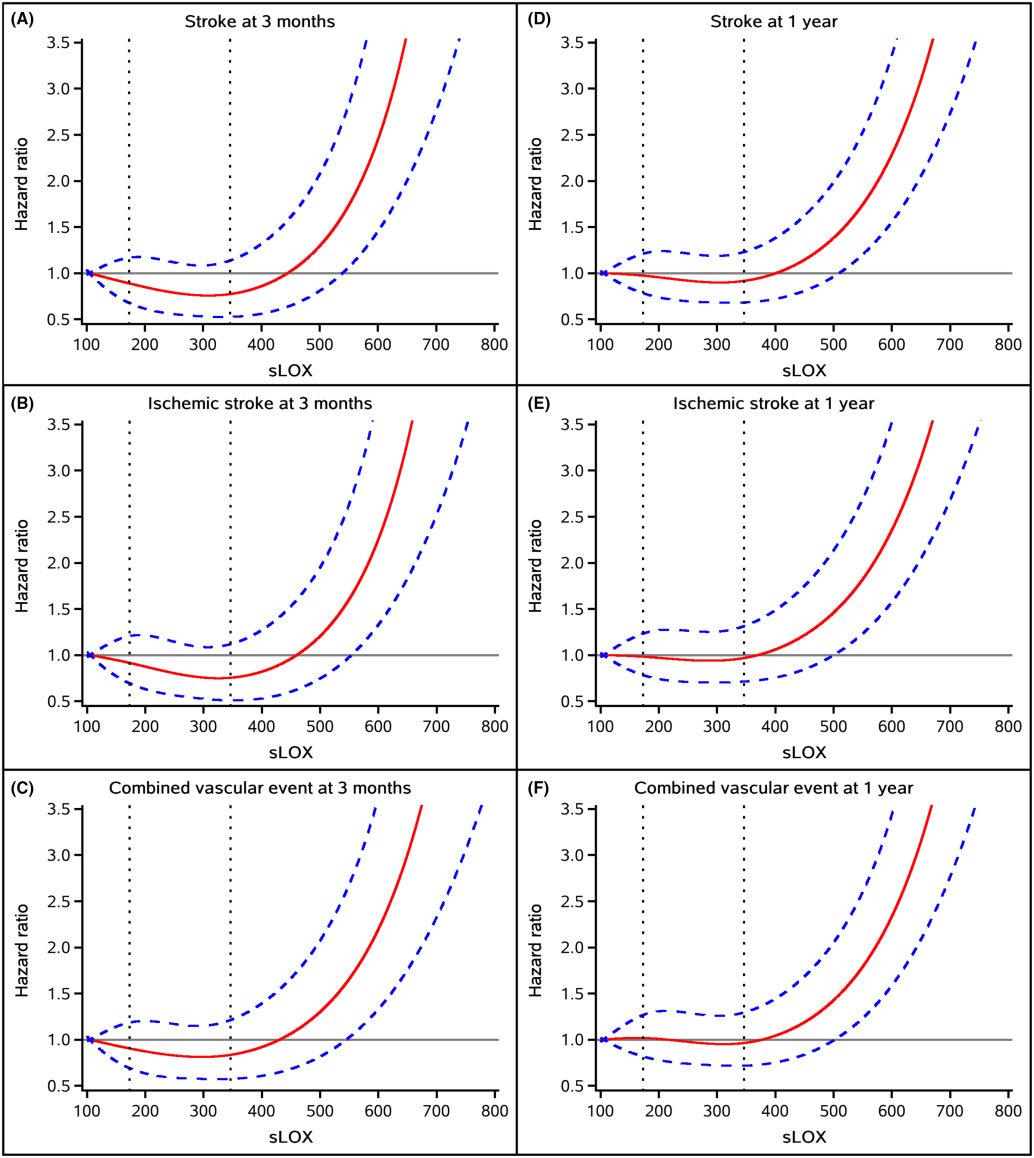
Association of sLOX‐1 levels with recurrent stroke, ischemic stroke, and combined vascular events within 3 months and 1 year. Abbreviations: sLOX‐1 = Soluble lectin‐like oxidized low‐density lipoprotein receptor‐1. Adjusted for body mass index, fasting blood glucose, high‐density lipoprotein cholesterol, NIHSS, history of stroke, heart failure, stroke subtype, TOAST, antiplatelet agents, and anticoagulant agents.

In the unadjusted and adjusted analysis (Table 2), a higher sLOX‐1 was significantly associated with higher odds for recurrent stroke within 3 months and 1 year follow‐up, the adjusted OR for the highest tertile vs lowest tertile of sLOX‐1 was 2.10 (95% CI, 1.40–3.16; *p* for trend < 0.0001), and 2.23 (95% CI, 1.61–3.08; *p* for trend < 0.0001), respectively. Similar results were observed for ischemic stroke (adjusted OR, 1.95; 95% CI, 1.28–2.96) and combined vascular events (adjusted OR, 1.95; 95% CI, 1.28–2.96) within 3 months and 1 year (adjusted OR with 95% CI was 2.30 [1.66–3.19] and 2.31 [1.64–3.24], respectively). Multivariable‐adjusted spline regression models showed J‐shaped associations between sLOX‐1 levels and the odds of recurrent stroke, ischemic stroke, and combined vascular events within 3 months and 1 year (Figure 2). Sensitivity analysis excluding recurrent stroke within the first month yielded the similar results (Table S3).

**TABLE 2:** Odds ratio (95% confidence interval) for outcomes according to sLOX‐1 tertiles

Outcomes	sLOX‐1 tertiles	Outcomes within 3 months			Outcomes within 1 year		
		Events, *n* (%)	Unadjusted	Adjusted	Events, *n* (%)	Unadjusted	Adjusted
Stroke	Tertile 1	79 (19.75)	Reference	Reference	109 (27.25)	Reference	Reference
	Tertile 2	63 (15.75)	0.79 (0.53–1.17)	0.74 (0.48–1.14)	108 (27.00)	0.99 (0.72–1.36)	0.93 (0.66–1.30)
	Tertile 3	115 (28.75)	2.13 (1.46–3.12)	2.10 (1.40–3.16)	183 (45.75)	2.34 (1.72–3.18)	2.23 (1.61–3.08)
	*p* for trend		<0.0001	<0.0001		<0.0001	<0.0001
Ischemic Stroke	Tertile 1	77 (19.25)	Reference	Reference	100 (25.00)	Reference	Reference
	Tertile 2	60 (15.00)	0.76 (0.51–1.14)	0.74 (0.47–1.15)	100 (25.00)	1.01 (0.72–1.40)	0.96 (0.67–1.37)
	Tertile 3	106 (26.50)	1.95 (1.33–2.88)	1.95 (1.28–2.96)	168 (42.00)	2.32 (1.69–3.20)	2.31 (1.64–3.24)
	*p* for trend		<0.0001	0.0012		<0.0001	<0.0001
Combined vascular events	Tertile 1	79 (19.75)	Reference	Reference	110 (27.50)	Reference	Reference
	Tertile 2	67 (16.75)	0.85 (0.57–1.25)	0.79 (0.51–1.22)	115 (28.75)	1.08 (0.79–1.48)	1.01 (0.72–1.41)
	Tertile 3	116 (29.00)	2.16 (1.48–3.16)	2.09 (1.39–3.15)	184 (46.00)	2.42 (1.78–3.30)	2.30 (1.66–3.19)
	*p* for trend		<0.0001	<0.0001		<0.0001	<0.0001

Abbreviations: sLOX‐1 = Soluble lectin‐like oxidized low‐density lipoprotein receptor‐1.

*Note*: adjusted for body mass index, fasting blood glucose, high‐density lipoprotein cholesterol, NIHSS, history of stroke, heart failure, stroke subtype, TOAST, antiplatelet agents, and anticoagulant agents.

### 3.3 Incremental predictive value of sLOX‐1

We evaluated whether sLOX‐1 would further increase the predictive value of conventional risk factors (Table 3). For recurrent stroke within 1 year months as the outcome of interest, the C statistics by the conventional model was significantly improved with the addition of sLOX‐1 (from 0.66 [95% CI, 0.62–0.69] to 0.76 [95% CI, 0.74–0.79]; *p* < 0.0001). Furthermore, the discriminatory power and risk reclassification also appeared to be substantially better (IDI 13.38% [95% CI, 11.21%–15.55%], *p* < 0.0001; NRI 55.39% [43.80%–66.98%], *p* < 0.0001). Similar results were found for ischemic stroke, combined vascular events, and when the time point was set up as 3 months.

**TABLE 3:** Reclassification and discrimination statistics for outcomes within 3 months and 1 year by sLOX‐1

	C statistics	*p* value	IDI	*p* value	NRI	*p* value
	Estimate (95% CI)		Estimate (95% CI), %		Estimate (95% CI), %	
Outcomes within 3 months						
Stroke						
Conventional model	0.69 (0.65–0.73)	<0.0001	Reference		Reference	
Conventional model + sLOX‐1	0.78 (0.74–0.81)		12.89 (10.20–15.58)	<0.0001	50.00 (35.42–64.58)	<0.0001
Ischemic stroke						
Conventional model	0.69 (0.65–0.73)	<0.0001	Reference		Reference	
Conventional model + sLOX‐1	0.77 (0.73–0.80)		11.19 (8.60–13.79)	<0.0001	46.28 (31.22–61.34)	<0.0001
Combined vascular events						
Conventional model	0.70 (0.66–0.74)		Reference		Reference	
Conventional model + sLOX‐1	0.78 (0.74–0.81)	<0.0001	11.49 (8.97–14.02)	<0.0001	46.26 (31.71–60.81)	<0.0001
Outcomes within 1 year						
Stroke						
Conventional model	0.66 (0.62–0.69)		Reference		Reference	
Conventional model + sLOX‐1	0.76 (0.74–0.79)	<0.0001	13.38 (11.21–15.55)	<0.0001	55.39 (43.80–66.98)	<0.0001
Ischemic stroke						
Conventional model	0.66 (0.63–0.70)		Reference		Reference	
Conventional model + sLOX‐1	0.76 (0.74–0.79)	<0.0001	12.92 (10.69–15.15)	<0.0001	52.32 (40.18–64.45)	<0.0001
Combined vascular events						
Conventional model	0.67 (0.64–0.70)		Reference		Reference	
Conventional model + sLOX‐1	0.77 (0.74–0.80)	<0.0001	13.08 (10.96–15.20)	<0.0001	56.63 (45.12–68.13)	<0.0001

Abbreviations: CI = confidence interval; IDI = integrated discrimination improvement; NRI = net reclassification index; sLOX‐1 = Soluble lectin‐like oxidized low‐density lipoprotein receptor‐1.

*Note*: adjusted for body mass index, fasting blood glucose, high‐density lipoprotein cholesterol, NIHSS, history of stroke, heart failure, stroke subtype, TOAST, antiplatelet agents, and anticoagulant agents.

### 3.4 Subgroup analyses

**FIGURE 3:**
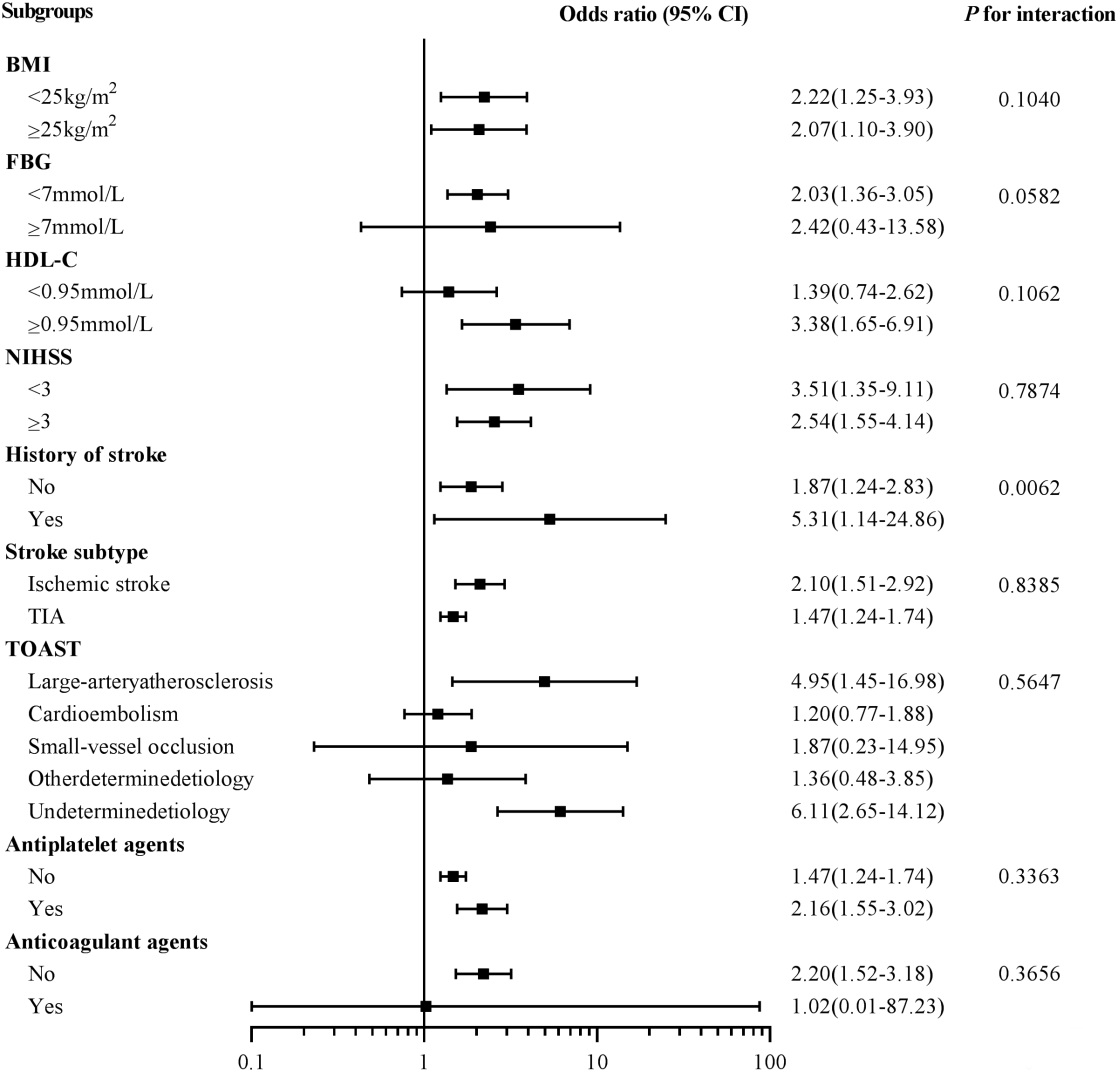
Subgroup analysis for the association between the highest tertile sLOX‐1 and recurrent stroke. Abbreviations: BMI = body mass index; CI = confidence interval; FBG = fasting blood glucose; HDL‐C = high density lipoprotein cholesterol; NIHSS = The National Institutes of Health Stroke Scale; sLOX‐1 = Soluble lectin‐like oxidized low‐density lipoprotein receptor‐1; TOAST = Trial of Org 10,172 in Acute Stroke Treatment. Adjusted for body mass index, fasting blood glucose, high‐density lipoprotein cholesterol, NIHSS, history of stroke, stroke subtype, TOAST, antiplatelet agents, and anticoagulant agents other than variables for stratification.

Results of subgroup analysis were presented in Figure 3. There was a significant interaction between history of stroke and sLOX‐1 in relation to recurrent stroke (*p* for interaction = 0.0062). The association between a higher sLOX‐1 level and recurrent stroke was more pronounced in patients with a history of stroke (OR, 5.31; 95% CI, 1.14–24.86) than those without a history of stroke (OR, 1.87; 95% CI, 1.24–2.83). In terms of other stratified variables, the association between sLOX‐1 and recurrent stroke was consistent across other subgroups, no significant interaction was observed (*p* for interaction > 0.05 for all).

## 4 DISCUSSION

The major finding of this nested case–control study was that a higher level of sLOX‐1 was independently associated with a higher risk of recurrent stroke, as well as ischemic stroke and combined vascular events, in patients with AIS or TIA within 3 months and 1 year follow‐up. Additionally, there was a significant interaction between history of stroke and sLOX‐1, the association was more pronounced in patients with a history of stroke than those without a history of stroke.

LOX‐1 as a receptor of oxLDL is expressed in atherosclerotic carotid arteries. There are several possible sources of sLOX‐1 in the circulation. It is tempting to assume that the main shedding of sLOX‐1 originates from the vasculature, studies on human and/or animal cell lines also have shown the expression of LOX‐1 in several nonvascular cells, including adipocytes, neuron, platelets, cardiomyocytes, as well as in renal and lung tissue during pathological condition.^14^, ^15^, ^16^ Although the basal expression of sLOX‐1 is low, it is induced by oxidative and inflammatory stimuli. Circulating LOX‐1 thus may reflect the levels of inflammatory response and oxidative stress in systemic vasculature. Oxidative stress is present within the first hours after stroke. It is a reasonable hypothesis that the elevated levels of sLOX‐1 correlated to inflammation and may be a contributor in the pathway to atherosclerosis, including stroke. Furthermore, the quantification of sLOX‐1 was fast, easy, and cost‐effective, it could be a potential biomarker to enhance our understanding of the pathogenesis of stroke and improve the risk stratification of recurrent stroke.

The role of sLOX‐1 as a potentially prognostic biomarker for risk stratification in ischemic heart disease is thoroughly evaluated. Several studies implied that sLOX‐1 has potential as a sensitive biomarker for acute coronary syndrome and appears to be significantly higher in acute coronary syndrome than in stable coronary artery disease.^17^ sLOX‐1 is reported to be associated with an increased risk of per‐procedural myocardial infarction in coronary artery disease patients undergoing elective percutaneous coronary intervention.^11^, ^18^, ^19^ sLOX‐1 is also related to the prognosis of myocardial infarction, recurrent MI and long‐term all‐cause mortality are higher among those with high sLOX‐1 compared with those with low sLOX‐1 levels.^20^ However, the clinical use of sLOX‐1 in ischemic stroke patients is elusive. Data from the National Cerebral and Cardiovascular Center and Oslo Cohort study showed that patients with stroke had a high level of sLOX‐1.^7^, ^11^, ^21^ A cohort study included 241 subjects (148 consecutive patients with acute ischemic stroke with the subtype of large‐artery atherosclerotic stroke and 93 non‐stroke controls) reported that serum sLOX‐1 level was an independent predictor of functional outcome in patients with large‐artery atherosclerotic stroke.^21^ Another Japanese community‐cohort study showed that sLOX‐1 levels have been used in a formula to calculate LOX index and this LOX index can predict ischemic stroke during follow‐up.^11^ However, the sample size in prior studies was small, which might lead to statistical bias. Additionally, evidence on the role of sLOX‐1 in the pathological progression of recurrent stroke among patients with stroke was limited.

To fill the knowledge gap, our present study with an age‐ and sex‐matched nested case–control design and enrolled 1200 patients with stroke to investigate the association of sLOX‐1 and recurrent stroke. In accordance with above findings, our study demonstrated that increased sLOX‐1 can independently predict the risk of recurrent stroke, there was a J‐shaped association between sLOX‐1 and recurrent stroke. The addition of sLOX‐1 to the conventional risk factors could increase the discriminatory power and risk reclassification for recurrent stroke. In addition, we found the association of sLOX‐1 and recurrent stroke was more pronounced in those with a history of stroke. Possible reason underlying this result may be that patients who had a history of stroke likely to have more atherosclerotic burden, thus a higher level of inflammatory response and oxidative stress in systemic vasculature cause by a higher level of sLOX‐1 may contribute more to the development of recurrent stroke. Our study provided evidence on the role of sLOX‐1 in the pathological pathway of stroke and supported the notion that history of stroke, as a predisposing factor to recurrent cerebral stroke, modified the the association between sLOX‐1 and recurrent stroke.

The potential mechanisms responsible for the association between sLOX‐1 and recurrent stroke are summarized as follows. First, sLOX‐1 is correlated to systematic inflammatory. sLOX‐1 is mainly expressed in endothelial cells but extends its expression to smooth muscle cells and macrophages in advanced lesions. Through the interaction with oxLDL, sLOX‐1 induces endothelial cells dysfunction, proliferation, and apoptosis of smooth muscle cells, accumulation of lipids in macrophages, formation of foam cell, and activation of platelet, resulting in a spectrum of proatherogenic cellular responses that facilitate atherosclerotic plaque initiation and progression.^14^, ^22^, ^23^, ^24^ Second, sLOX‐1 is associated with atherosclerotic factors and atherosclerosis. In vivo studies, LOX‐1 expression is increased in experimental and clinical studies of hypertension, hyperlipidemia, type 2 diabetes mellitus, obesity, and carotid artery atherosclerosis.^25^, ^26^, ^27^, ^28^, ^29^ Additionally, it was found that LOX‐1 is regulated by vasoconstrictive peptides, pro‐inflammatory cytokines, and other pathophysiological stimuli associated with atherosclerosis, which may play an important role in the recurrence of stroke.^29^, ^30^, ^31^, ^32^

Our study has several limitations. First, this is a nested case–control study, with observations relation to prognostic value of sLOX‐1 in recurrent stroke being particularly susceptible to confounding by selection bias. Second, changes in sLOX‐1 levels before and after the onset of stroke have not been examined. Notably, elevated sLOX‐1 levels were independent of the time since relevant cerebral symptoms and were prolonged for at least 3 months.^11^ Third, the study was conducted among Chinese, which may limit the generalization of the findings.

## 5 CONCLUSIONS

In conclusion, our study showed that elevated sLOX‐1 levels could independently predict recurrent stroke in patients with AIS or TIA. From a public health viewpoint, sLOX‐1 may provide new insights into the pathophysiology of stroke and may be a potential biomarker to improve risk stratification for recurrent stroke.

## AUTHORS CONTRIBUTIONS

X.M. and Y.W. contributed to the conception and design of the study; A.W., X.T., and X.J. contributed to manuscript drafting; A.W., X.T., and Q.X. contributed to the statistics analysis; H.L. and P.C. contributed to the acquisition of data; all authors contributed to critical revisions of the manuscript.

## CONFLICT OF INTEREST

All the authors have no conflict of interest.

## FUNDING INFORMATION

This study is supported by grants from the Capital's Funds for Health Improvement and Research (2020–1‐2041), Chinese Academy of Medical Sciences Innovation Fund for Medical Sciences (2019‐I2M‐5‐029), National Natural Science Foundation of China (81,870,905, U20A20358, 82,111,530,203), and Beijing Municipal Administration of Hospitals Incubating Program (PX2020021). The funder has no role in study design, data collection, data analysis, manuscript preparation, and/or publication decisions.

## Supporting information


Appendix S1

## Data Availability

Data are available to researchers on request for purposes of reproducing the results or replicating the procedure by directly contacting the corresponding author.
