# Supplementary material for: Soluble lectin‐like oxidized low‐density Lipoproteinreceptor‐1 and recurrent stroke: A nested case–control study
Source: CNS Neurosci Ther. 2022 Jul 31;28(12):2001–10. doi: 10.1111/cns.13932 (PMC9627350; doi:10.1111/cns.13932)
Supplement: Supplementary file 1 — Appendix S1 [file CNS-28-2001-s001.docx]

**Supplemental materials**

Table S1. Comparison of baseline characteristics between enrolled patients and excluded patients

| Characteristics | Excluded  (n=1073) | Enrolled  (n=400) | *P* value |
| --- | --- | --- | --- |
| Age, y | 64(56-72) | 64(55-73) | 0.8851 |
| Men, n (%) | 714 (66.54) | 266 (66.50) | 0.9878 |
| Body mass index, kg/m^2^ | 24.44(22.58-26.57) | 24.92(22.81-27.25) | 0.0622 |
| Medical History, n (%) |  |  |  |
| Hypertension | 697 (64.96) | 265 (66.25) | 0.6431 |
| Diabetes mellitus | 278 (25.91) | 119 (29.75) | 0.1395 |
| Dyslipidemia | 77 (7.18) | 36 (9.00) | 0.2421 |
| Stroke or TIA | 349 (32.53) | 115 (28.75) | 0.1653 |
| Atrial fibrillation/flutter | 102 (9.51) | 35 (8.75) | 0.6568 |
| Peripheral vascular disease | 11 (1.03) | 2 (0.50) | 0.3378 |
| Heart failure | 14 (6.70) | 3 (4.76) | 0.5778 |
| Stroke type/Subtype, n (%) | | | |
| Ischemic stroke | 1034 (96.37) | 390 (97.50) | 0.2801 |
| TIA | 39 (3.63) | 10 (2.50) |  |
| TOAST, n (%) |  |  |  |
| Large-artery atherosclerosis | 349 (32.53) | 140 (35.00) | 0.4589 |
| Cardioembolism | 72 (6.71) | 34 (8.50) |  |
| Small-vessel occlusion | 175 (16.31) | 59 (14.75) |  |
| Other determined etiology | 13 (1.21) | 7 (1.75) |  |
| Undetermined etiology | 464 (43.24) | 160 (40.00) |  |
| Medication in hospital, n (%) | | | |
| Cholesterol-lowering agents | 1027 (96.25) | 383 (95.99) | 0.8164 |
| Antihypertensive agents | 536 (50.23) | 195 (48.87) | 0.6425 |
| Hypoglycemic agents | 297 (27.84) | 131 (32.83) | 0.0611 |
| Antiplatelet agents | 1014 (95.03) | 377 (94.49) | 0.6725 |
| Anticoagulant agents | 163 (15.28) | 69 (17.29) | 0.3464 |
| NIHSS score on admission | 4(2-6) | 4(2-7) | 0.1138 |
| Laboratory tests |  |  |  |
| Total cholesterol, mmol/L | 4.05(3.34-4.88) | 3.95(3.23-4.65) | 0.0798 |
| LDL-C, mmol/L | 2.39(1.80-3.13) | 2.29(1.74-2.95) | 0.0916 |
| HDL-C, mmol/L | 0.93(0.76-1.13) | 0.93(0.78-1.09) | 0.9706 |
| Triglyceride, mmol/L | 1.34(1.02-1.88) | 1.31(1.00-1.82) | 0.4547 |
| Fasting blood glucose, mmol/L | 5.76(5.07-7.40) | 5.81(5.08-7.65) | 0.7314 |

NIHSS=The National Institutes of Health Stroke Scale; TIA=transient Ischemic Attack; TOAST= Trial of Org 10172 in Acute Stroke Treatment.

Table S2. Baseline characteristics according to tertiles of sLOX-1 levels

| Characteristics | Tertiles of sLOX-1 | | | *P* value |
| --- | --- | --- | --- | --- |
|  | Tertiles 1 | Tertiles 2 | Tertiles 3 |  |
| Age, y | 63.5(55-73) | 64(55-72) | 64(57-73) | 0.8387 |
| Men, n (%) | 257 (64.25) | 263 (65.75) | 278 (69.50) | 0.2690 |
| Body mass index, kg/m^2^ | 24.29(22.48-26.77) | 24.49(22.53-26.57) | 24.77(22.86-26.73) | 0.2108 |
| Time from onset of symptoms to  admission ≥24 hours, n (%) | 169 (42.25) | 162 (40.50) | 151 (37.75) | 0.4247 |
| Medical History, n (%) |  |  |  |  |
| Hypertension | 258 (64.50) | 263 (65.75) | 253 (63.25) | 0.7611 |
| Diabetes mellitus | 136 (34.00) | 103 (25.75) | 105 (26.25) | 0.0152 |
| Dyslipidemia | 41 (10.25) | 34 (8.50) | 29 (7.25) | 0.3174 |
| Stroke or TIA | 100 (25.00) | 94 (23.50) | 110 (27.50) | 0.4217 |
| Atrial fibrillation/flutter | 35 (8.75) | 36 (9.00) | 29 (7.25) | 0.6256 |
| Peripheral vascular disease | 4 (1.00) | 2 (0.50) | 7 (1.75) | 0.2282 |
| Heart failure | 0 (0.00) | 6 (10.34) | 4 (6.25) | 0.0396 |
| Stroke type/Subtype, n (%) |  |  |  |  |
| Ischemic stroke | 376 (94.00) | 374 (93.50) | 379 (94.75) | 0.7524 |
| TIA | 24 (6.00) | 26 (6.50) | 21 (5.25) |  |
| TOAST, n (%) |  |  |  |  |
| Large-artery atherosclerosis | 110 (27.50) | 91 (22.75) | 143 (35.75) | 0.0013 |
| Cardioembolism | 31 (7.75) | 37 (9.25) | 26 (6.50) |  |
| Small-vessel occlusion | 68 (17.00) | 71 (17.75) | 76 (19.00) |  |
| Other determined etiology | 1 (0.25) | 4 (1.00) | 6 (1.50) |  |
| Undetermined etiology | 190 (47.50) | 197 (49.25) | 149 (37.25) |  |
| Medication in hospital, n (%) |  |  |  |  |
| Cholesterol-lowering agents | 387 (96.99) | 383 (95.75) | 382 (95.50) | 0.5079 |
| Antihypertensive agents | 179 (44.86) | 198 (49.50) | 198 (49.50) | 0.3175 |
| Hypoglycemic agents | 145 (36.34) | 105 (26.25) | 112 (28.00) | 0.0040 |
| Antiplatelet agents | 388 (97.24) | 380 (95.00) | 388 (97.00) | 0.1735 |
| Anticoagulant agents | 41 (10.28) | 58 (14.50) | 51 (12.75) | 0.1932 |
| NIHSS score on admission | 3(2-6) | 4(2-6) | 4(2-6) | 0.1599 |
| Laboratory tests |  |  |  |  |
| Total cholesterol, mmol/L | 3.87(3.43-4.61) | 3.86(3.29-4.69) | 3.92(3.23-4.52) | 0.3838 |
| LDL-C, mmol/L | 2.20(1.80-2.87) | 2.24(1.73-2.92) | 2.24(1.74-2.88) | 0.9717 |
| HDL-C, mmol/L | 0.96(0.81-1.16) | 0.97(0.79-1.15) | 0.92(0.77-1.08) | 0.0102 |
| Triglyceride, mmol/L | 1.36(1.07-1.9) | 1.35(1.02-1.76) | 1.32(0.97-1.77) | 0.1948 |
| Fasting blood glucose, mmol/L | 5.60(4.89-7.64) | 5.62(4.83-6.89) | 5.51(4.98-7.10) | 0.5300 |
| eGFR, mL/min/1.73 m^2^ | 92.36(81.95-101.07) | 90.76(79.06-101.72) | 91.76(80.8-99.28) | 0.7458 |
| Hs-CRP, mg/dL | 2.95(1.11-5.31) | 3.38(1.95-7.10) | 3.40(1.51-8.00) | 0.3430 |
| sLOX, ng/L | 105.03(68.10-132.81) | 247.12(210.16-295.24) | 485.70(413.58-647.03) | <0.0001 |

eGFR=estimated glomerular filtration rate; hs-CRP, high sensitivity C-reactive protein; NIHSS=The National Institutes of Health Stroke Scale; sLOX-1=Soluble lectin-like oxidized low-density lipoprotein receptor-1; TIA=transient Ischemic Attack; TOAST= Trial of Org 10172 in Acute Stroke Treatment.

Table S3. Sensitivity analysis excluding recurrent stroke occurred within the first month (N=642)

| Outcomes | Slox-1 tertiles | Outcomes within 90 days | | |  | Outcomes within 1 year | | |
| --- | --- | --- | --- | --- | --- | --- | --- | --- |
|  |  | Events, n (%) | Unadjusted | Adjusted |  | Events, n (%) | Unadjusted | Adjusted |
| Stroke | Tertile 1 | 19(9.13) | Reference | Reference |  | 49 (23.56) | Reference | Reference |
|  | Tertile 2 | 16(7.62) | 0.97(0.44-2.16) | 1.10(0.46-2.60) |  | 61 (29.05) | 1.32(0.84-2.06) | 1.28(0.80-2.06) |
|  | Tertile 3 | 36 (16.07) | 3.48(1.58-7.68) | 3.72(1.54-9.00) |  | 104(46.43) | 2.97(1.92-4.60) | 2.82(1.78-4.47) |
|  | *P* for trend |  | 0.0017 | 0.0030 |  |  | <0.0001 | <0.0001 |
| Ischemic Stroke | Tertile 1 | 18(8.65) | Reference | Reference |  | 41 (19.71) | Reference | Reference |
|  | Tertile 2 | 16(7.62) | 1.11(0.49-2.52) | 1.30(0.53-3.16) |  | 56 (26.67) | 1.54(0.95-2.49) | 1.56(0.93-2.61) |
|  | Tertile 3 | 34 (15.18) | 3.50(1.56-7.86) | 3.74(1.50-9.33) |  | 96 (42.86) | 3.39(2.12-5.43) | 3.38(2.04-5.61) |
|  | *P* for trend |  | 0.0021 | 0.0042 |  |  | <0.0001 | <0.0001 |
| Combined vascular events | Tertile 1 | 19(9.13) | Reference | Reference |  | 50 (24.04) | Reference | Reference |
|  | Tertile 2 | 17(8.10) | 1.05(0.48-2.32) | 1.20(0.52-2.79) |  | 64 (30.48) | 1.45(0.92-2.26) | 1.40(0.87-2.24) |
|  | Tertile 3 | 36 (16.07) | 3.36(1.55-7.32) | 3.72(1.55-8.91) |  | 104(46.43) | 3.10(1.99-4.81) | 2.94(1.85-4.68) |
|  | *P* for trend |  | <0.0001 | <0.0001 |  |  | <0 .0001 | <0.0001 |

Abbreviations: sLOX-1, Soluble lectin-like oxidized low-density lipoprotein receptor-1

Adjusted for body mass index, fasting blood glucose, high-density lipoprotein cholesterol, NIHSS, history of stroke, heart failure, stroke subtype, TOAST, antiplatelet agents, and anticoagulant agents.
